# Musculoskeletal pain in children and adolescents

**DOI:** 10.1590/bjpt-rbf.2014.0149

**Published:** 2016-02-16

**Authors:** Steve J. Kamper, Nicholas Henschke, Lise Hestbaek, Kate M. Dunn, Christopher M. Williams

**Affiliations:** 1 The George Institute, University of Sydney, Australia; 2 Institute of Public Health, University of Heidelberg, Heidelberg, Germany; 3 Department of Sport Science and Clinical Biomechanics, University of Southern Denmark, Odense, Denmark; 4 Arthritis Research UK Primary Care Centre, Institute for Primary Care and Health Sciences, Keele University, Keele, UK; 5 Hunter Medical Research Institute, School of Medicine and Public Health, University of Newcastle, Newcastle, Australia; 6 Hunter New England Population Health, Hunter New England Local Health District, Australia

**Keywords:** children, adolescents, musculoskeletal pain, burden, epidemiology

## Abstract

**Introduction:**

Musculoskeletal (MSK) pain in children and adolescents is responsible for substantial personal impacts and societal costs, but it has not been intensively or systematically researched. This means our understanding of these conditions is limited, and healthcare professionals have little empirical evidence to underpin their clinical practice. In this article we summarise the state of the evidence concerning MSK pain in children and adolescents, and offer suggestions for future research.

**Results:**

Rates of self-reported MSK pain in adolescents are similar to those in adult populations and they are typically higher in teenage girls than boys. Epidemiological research has identified conditions such as back and neck pain as major causes of disability in adolescents, and in up to a quarter of cases there are impacts on school or physical activities. A range of physical, psychological and social factors have been shown to be associated with MSK pain report, but the strength and direction of these relationships are unclear. There are few validated instruments available to quantify the nature and severity of MSK pain in children, but some show promise. Several national surveys have shown that adolescents with MSK pain commonly seek care and use medications for their condition. Some studies have revealed a link between MSK pain in adolescents and chronic pain in adults.

**Conclusion:**

Musculoskeletal pain conditions are often recurrent in nature, occurring throughout the life-course. Attempts to understand these conditions at a time close to their initial onset may offer a better chance of developing effective prevention and treatment strategies.

## Bullet points

- The prevalence of MSK pain approaches adult levels by the end of adolescence.
- Persistent adolescent MSK pain is a risk factor for chronic pain in adulthood.
- MSK pain has substantial impacts in up to 1/4 of cases.
- The relationship of other adverse health risk factors and MSK pain is unclear.
- There is little research to inform clinical management of childhood MSK pain.

## Background

Research by the World Health Organisation has brought the enormous global burden of musculoskeletal (MSK) pain into focus. Low back pain (LBP), neck pain and other musculoskeletal disorders were ranked numbers 1, 4 and 10 respectively, among all health conditions for years lived with disability. These conditions were also identified as the main drivers of the increase in years lived with disability over the past 20 years^1^. Although the epidemiology, burden and treatment of MSK pain in adults has been the subject of considerable research efforts, the same is not true for children. The dearth of clinical research relevant to children and adolescents has been highlighted by several authors^2^ ^-^ ^5^. The purpose of this paper is to outline the current state of understanding with respect to MSK pain in people under the age of 18 years.

The lack of research into MSK pain in children is of concern for a number of reasons. There is emerging evidence that children, especially adolescents who report persistent pain, are at increased risk of chronic pain as adults^6^ ^-^ ^8^. This is important since adults with persistent pain endure the bulk of the individual and societal burden of painful conditions^9^. Further, many MSK conditions follow a long-term pattern of recurring exacerbations and remissions, with the most consistent predictor of a new episode being experience of a previous episode^10^. Given that the rise in prevalence of MSK conditions occurs in adolescence, it may be necessary to investigate the condition in this stage of life (or earlier) to identify the initial onset. Understanding factors surrounding the initial onset offers the best chance of developing successful treatments, and is fundamental to any efforts at primary prevention. Finally, clinicians who care for children with MSK conditions have little research evidence to inform their clinical decisions, since clinical practice guidelines, such as those for the treatment of LBP, either specifically exclude children or base their recommendations on adult research^11^.

There is a question regarding the extent to which research conducted in adults can be generalised to children. Pain, in particular chronic pain is currently conceptualised within a biopsychosocial model^12^. Thus the experience of pain is influenced by physical factors e.g. anatomical pathology and physiological process; psychological factors e.g. mood, cognitions and beliefs; and social factors e.g. relationships, social environment and culture. In each of these domains there are important differences between children and adults. Childhood and adolescence is a time of growth of the MSK system with changes in structural properties, biomechanics and motor control before the system stabilises in adulthood. Similarly, substantial cognitive and emotional development occurs during this time and hormonal changes regulate mood and emotions differently to adulthood. Finally, social relationships, expectations and environments experienced by children are distinct from those of adults^13^. These differences provide good reasons to consider that the pain experience may be different.

Providing direction to those responsible for the care of children with MSK conditions will be a key function of research efforts in the area. Balancing the competing needs of early identification and appropriate management of those who need care, and reassurance and avoidance of ‘medicalization’ of transient aches and pains is a difficult task. Yet it is critical; over-investigation and over-treatment of MSK pain results in a large burden on overloaded healthcare systems, and can also negatively impact individual patient outcomes^14^.

For the purposes of this review we focus on regional pain conditions e.g. non-specific neck or back pain, joint pain including hip, knee, shoulder and elbow pain. We do not discuss pain associated with congenital or systemic diseases, such as hip dysplasia, juvenile arthritis or scoliosis. We also exclude pain resulting from frank injuries e.g. anterior cruciate ligament rupture, ankle sprain, fractures and pain following surgical interventions. We collected evidence mainly from systematic reviews that describe the epidemiology, risk and prognostic factors, burden, and treatment of MSK pain in people under 18 years old. Where possible we have looked to studies published since 2000. To supplement the systematic reviews we included data from well-conducted cohort studies and surveys.

## Incidence and prevalence

Numerous cohorts and surveys have included questions about MSK pain, and have estimated the prevalence of conditions in children and adolescents. Unfortunately, methodological features and reporting methods make synthesis of the estimates difficult. Along with case definitions, there is heterogeneity with respect to the prevalence period; where possible we report 1-month prevalence to facilitate comparison. A further source of heterogeneity is due to different aged children included in the various studies.

**Figure 1:**
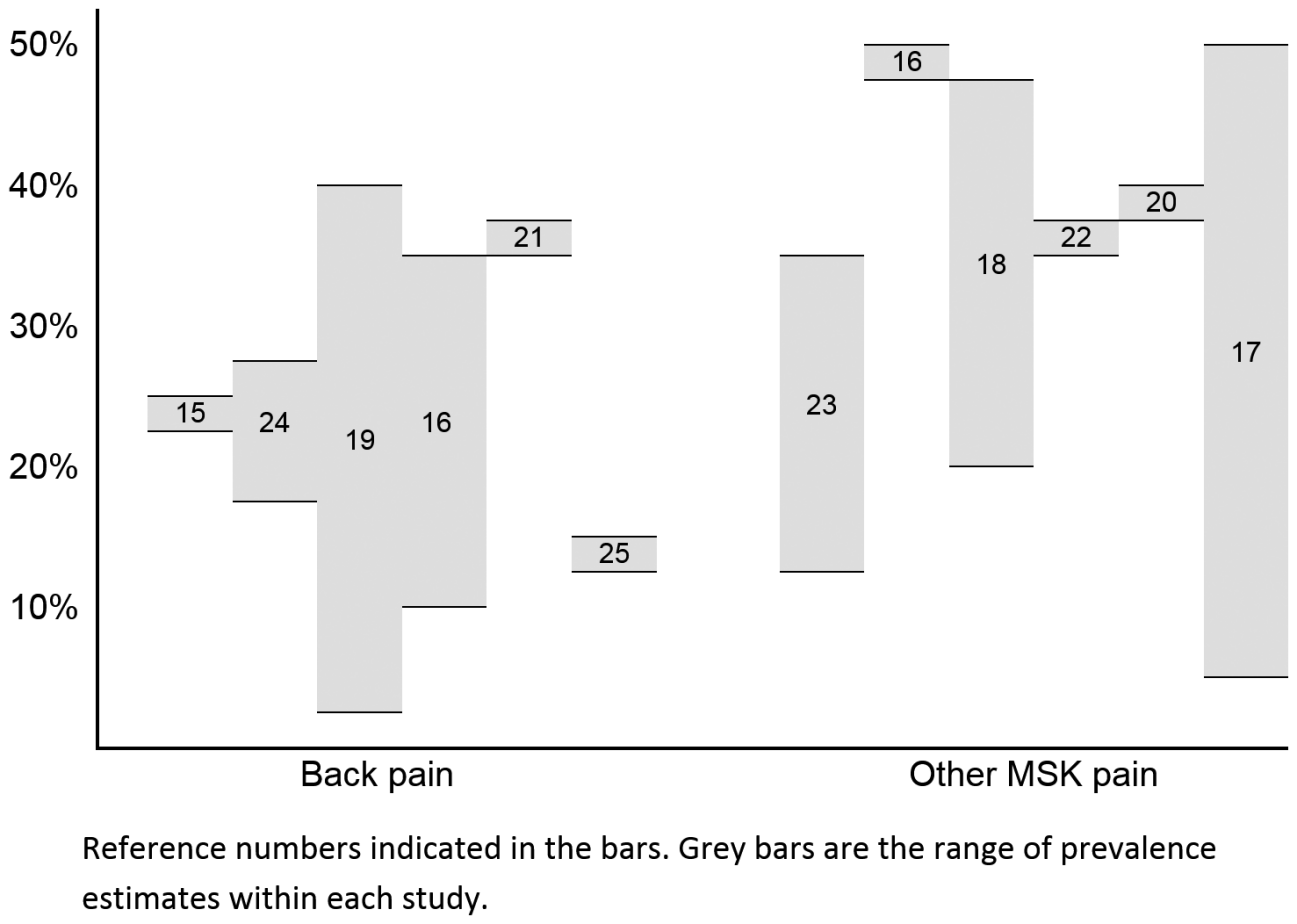
Range of prevalence estimates of MSK pain in adolescents reported by different studies. Reference numbers indicated in the bars. Grey bars are the range of prevalence estimates within each study.

A recent, comprehensive systematic review investigating the prevalence of LBP in all age groups noted that prevalence during adolescence approached that in the adult years^15^. Notwithstanding these methodological and population inconsistencies, approximately one third of adolescents report MSK pain on a monthly basis. Estimates from recent reviews and large, well-conducted cohort studies are presented in Figure 1 ^15^ ^-^ ^25^.

There are fewer studies that report the incidence of MSK pain in children, possibly due to logistical difficulty of conducting these studies. A systematic review reported 1-year incidence in the range of 18-33% for LBP and 28% for neck pain^16^, another reported 1-year incidence rates for mid-back pain in the range of 15-35%^23^.

Generally, studies reported higher prevalence and incidence rates in adolescent girls than boys^13^ and a sharp rise in rates from childhood to adolescence^26^ ^,^ ^27^, the boundary being at approximately 10-12 years. Differences in the timing and nature of pubertal development between the sexes^28^ ^-^ ^30^ has been proposed as an explanation, but as yet this theory has not been confirmed. See Box 1 for an overview of issues related to incidence and prevalence.

**Box 1:** Prevalence.

What we know • Up to one third of adolescents report MSK pain monthly or more. • Prevalence of MSK pain rises quickly from childhood to adolescence. • Adolescent girls more commonly report MSK pain than boys. What we need to know • What is the prevalence of consequential MSK pain in children and adolescents?

## Impact

Despite a view that non-specific MSK pain in children is often of little consequence, the global disability burden is large. Data from the WHO Global Burden of Disease study shows that LBP is responsible for the 2^nd^ most years lived with disability for 15-19 year olds of any health condition and neck pain ranks 8^th^. These are both higher than well-recognised adolescent public health problems such as asthma, alcohol and drug use and road injury^31^. This burden stands in stark contrast to the volume of research aimed at describing the consequences of MSK pain in children^32^. Of the longitudinal research that has been done, few studies follow-up more regularly than once per year, which means that detailed information about course and consequences of MSK pain is limited^33^.

**Figure 2:**
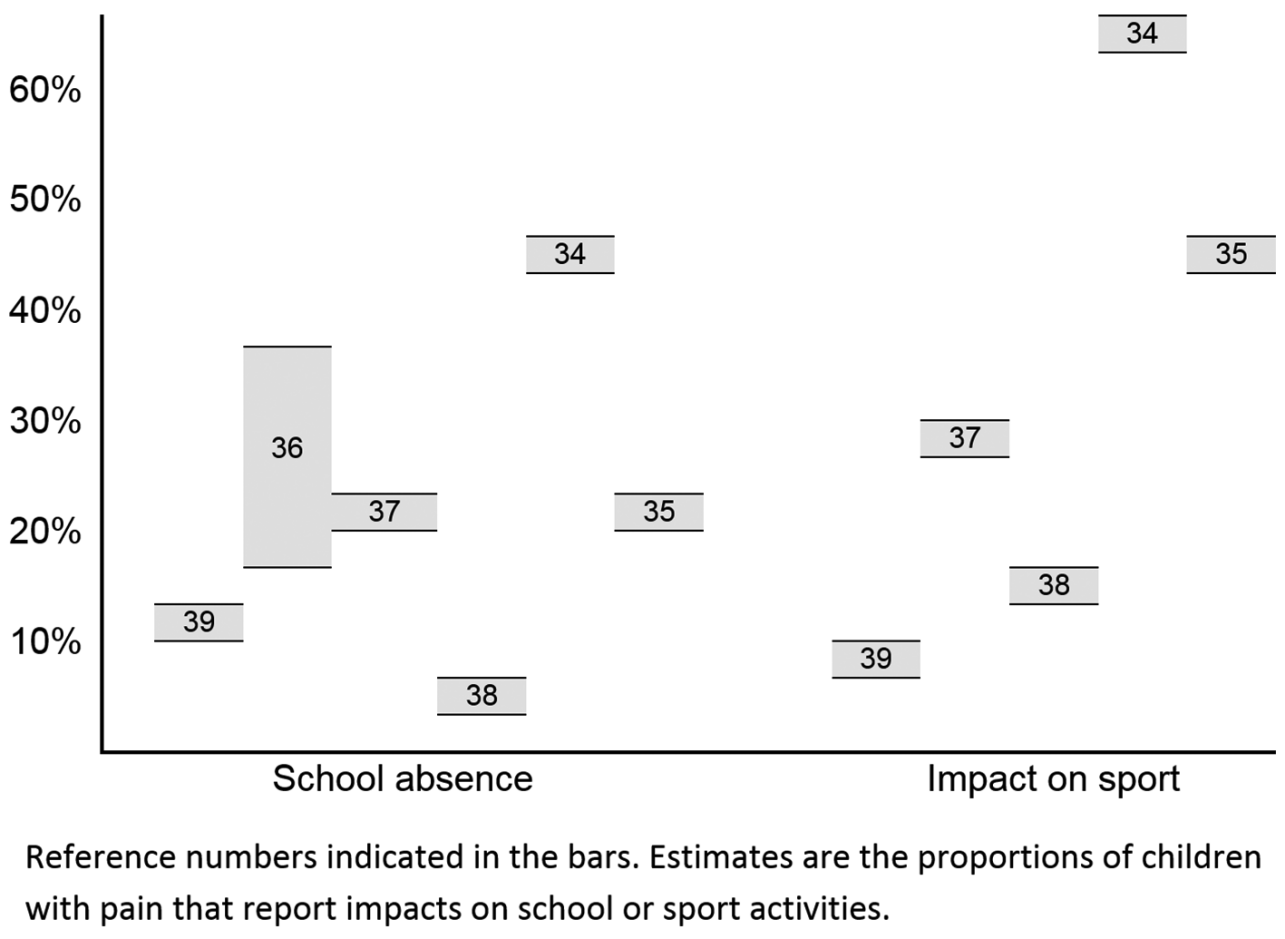
Estimates of the proportions of children with pain that report impacts on school or sport activities. Reference numbers indicated in the bars. Estimates are the proportions of children with pain that report impacts on school or sport activities.

School and school-related activities form major components of life and have substantial impacts on the development and future opportunities of children and adolescents. Similarly, involvement in sporting activities and general physical activity has physical, mental and social consequences that extend into adulthood. As such it is important that the impact of pain on participation in these areas is well-described and understood. Surveys that have been conducted suggest that a substantial minority of adolescents with MSK pain experience significant impacts on their lives. Estimates of proportions reporting these impacts are summarised in Figure 2 ^34^ ^-^ ^39^.

The association between MSK pain and physical activity is difficult to unravel. There are plausible theories to explain a causal relationship in both directions, and individual studies report conflicting results regarding the existence and strength of a relationship. A systematic review of 8 studies investigating the relationship between LBP and physical activity reported positive, negative and non-existent associations^40^. A more recent Danish study found no associations between spinal pain and objectively measured physical activity, either in cross-sectional or longitudinal analyses^41^ and a multi-national survey of 400,000 11-15 years olds found that the presence of LBP was generally associated with reduced physical activity, but the size of the relationship was small^42^. See Box 2 for an overview of issues related to impact.

**Box 2:** Impact.

What we know • A substantial minority of adolescents have disabling consequences due to MSK pain. • Of those that report MSK pain, up to a quarter may miss school or sport due to their condition. What we need to know • What is the clinical course of MSK pain over time? • What is the nature of the interaction between sport participation, physical activity and MSK pain?

## Risk and prognostic factors

Several cross-sectional and longitudinal studies report associations between back pain and psychological distress (anxiety and depression). Relationships between pain and mechanical load e.g. school bag weight, high BMI, are less clear. For instance, of 1,046 11-14 year olds, children who reported high levels of psychosocial difficulties at baseline were more likely (RR: 1.6) to develop LBP one year later, but there was no association between LBP and bag weight, BMI or sedentary levels^43^. Similarly, physical activity at baseline did not predict pain 12 months^43^ or 3 years later^44^. Other cohorts have found relationships between pain and: behavioural (sleep patterns, attention disorders)^45^ ^-^ ^47^, social^48^, and physical factors (headache, other illness, hypermobility, muscular tightness)^47^ ^,^ ^49^ ^,^ ^50^. A cohort of 377, 12-14 year old adolescents found high growth rate, smoking, muscle tightness and part-time work increased risk of back pain 12 months later^50^. Another longitudinal study following 1,773 15-19 years olds found that sleep quantity and quality predicted low back, neck and shoulder pain (OR range 4.4 to 2.2) 2 years later^45^.

While the majority of these studies aim to define risk factors which predict the development of musculoskeletal pain, inconsistent case definitions and reporting of covariates make interpretation difficult. Importantly, many studies do not control for previous history of pain, making it difficult to distinguish between risk factors and prognostic factors. Further, studies often use broad definitions of an ‘episode’ of pain, with no distinction between transient episodes and long term or consequential pain. As a consequence it is unclear whether individual factors predispose to pain, lead to a poor outcome from ‘an episode’ or are consequences of the condition.

This relationship with other adverse health risk factors suggests MSK pain may have an important role in shaping health in adulthood. Several studies have shown predictive longitudinal relationships between back pain in adolescents and factors such as smoking^51^ ^-^ ^53^, poor mental health, overweight^46^ ^,^ ^54^ ^,^ ^55^, inactivity and poor sleep^45^. Data from a prospective cohort of overweight children suggests an association between LBP and obesity and subsequent inactivity, with inactivity reinforcing further weight gain and further pain^54^. Another longitudinal study found that back pain at 14 years predicted smoking at 17^51^. It is likely the combination of low back pain and associated health risks contribute to a series of reciprocal events contributing to higher risk of pain, health risk behaviours and poorer general health. As poor lifestyle habits developed in adolescence are known to track into adulthood^56^, and pain is related to the development of adverse health risk factors, pain may be a contributor to the onset of lifelong chronic disease and mortality^57^. See Box 3 for an overview of issues related to risk and prognostic factors.

**Box 3:** Risk and prognostic factors.

What we know • Report of MSK pain is commonly associated with psychological distress. • Adolescents with MSK pain are at higher risk of also reporting adverse health risk factors such as smoking, overweight, poor mental health and poor sleep. What we need to know • What are the factors related to first onset of MSK that may form targets for primary prevention, as opposed to treatment or secondary prevention? • What is the role of MSK pain in the constellation of adverse health risk factors that place adolescents at higher risk of chronic disease in adulthood?

## Care-seeking, treatment, and prevention

In order to fully understand the public health impact of musculoskeletal pain in children and adolescents, it is important to understand care-seeking patterns, as visits to health care providers contribute to the societal burden and cost of MSK pain. A number of studies have been performed to estimate the proportion of children and adolescents who seek care as a result of MSK pain. In most cases the numbers are large and there exists a significant risk of poor outcome due to a lack of evidence-based treatments and clinical guidelines.

One study in a primary care setting in Spain reported that MSK pain is responsible for approximately 6% of the primary care visits of children between 3 and 14 years, and more than 10% of visits by adolescents^58^. In Australia, MSK problems in children and adolescents are managed by general practitioners at a rate of 5.8 per 100 encounters^59^. Upper and lower limb conditions were the most common, followed by spine and trunk. This can be extrapolated to 880,000 child or adolescent MSK problems managed by GPs per year in Australia^59^. In the United Kingdom, 24% of children with LBP reported visiting a doctor in the past year for the condition^60^, in Finland the figure is 16%^61^ and from a sample in Australia; 37.6%^35^.

The most common drivers of care-seeking for MSK pain appear to be high pain intensity and activity limitation^61^ ^,^ ^62^. There appears to be little difference in the care-seeking rate between boys and girls and the rate of MSK problems managed increases significantly with age^59^ ^-^ ^61^. In Denmark, care-seeking for back pain was found to be uncommon in children and much less common than the back pain prevalence (6% vs. 33%)^63^. By the age of 15, this gap had decreased (34% vs. 48%), which could indicate that either the symptoms become more bothersome or that the pain is not taken seriously by the parents until the child is older^63^. This highlights the need for studies on MSK pain to describe their findings within limited and discrete age ranges.

Medication is frequently prescribed and appears to be more often prescribed to older girls and those with spine and trunk conditions than those with upper and lower limb problems^59^. Emerging research has highlighted problems with unrestricted use of medications in younger age groups; adolescents with recurrent pain are more likely to use medicines also for non-pain conditions, such as nervousness and sleep difficulties^64^. In a recent systematic review of conservative interventions for low back pain in children and adolescents, only four randomised trials of treatment and eleven of prevention were identified^5^. The review suggested that supervised exercise has a large effect on pain compared to no treatment, but evidence quality was low, there was not enough good quality information to make conclusions regarding preventative interventions. See Box 4 for an overview of issues related to care-seeking, treatment and prevention.

**Box 4:** Care-seeking, treatment and prevention.

What we know • A substantial minority of adolescents with MSK pain seek care for their condition. What we need to know • How to identify the adolescents that need referral and management, and those that need reassurance and encouragement to continue normal activities. • How effective are simple treatments delivered in primary care for adolescents with MSK pain? • Can preventive interventions reduce the impacts of MSK pain in children and adolescents?

## Recommendations for future research

### Life-course approach to understanding MSK pain

The experience of musculoskeletal pain in children obviously has consequences for them at the time, but it may be even more important in their future. Life-course epidemiology provides a framework to investigate the effect of early life and childhood factors on health in later life as adults^65^ ^,^ ^66^. Using a life-course approach has led to significant improvements in understanding of respiratory conditions and cardiovascular disease, has promise in the study of back pain^67^ ^,^ ^68^, and may well be beneficial to studying musculoskeletal pain in general. There is already some evidence that the experience of pain among children is associated with the experience of pain among adults^8^, but the pathways and mechanisms for these associations are unknown. It is possible that a physiological, psychological or behavioural trigger is set when a child has a particular painful experience, which predisposes them to pain as an adult. There may also be an underlying genetic predisposition towards experiencing pain, which could increase the likelihood of pain episodes in both childhood and adulthood: this is supported by evidence for genetic influences on MSK pain^69^. Using life-course epidemiology as a framework for studying musculoskeletal conditions gives scope for a wide range of studies, and potential for far greater understanding of how and why musculoskeletal conditions occur over the life course. See Box 5 for an overview of issues related to the life-course approach to research.

**Box 5:** Life-course approach.

What we know • MSK pain conditions typically follow a recurrent course with initial onset in adolescence. • Life-course epidemiology has improved understanding of other conditions with a similar lifetime profile. What we need • Studies that set MSK pain in this framework.

### Measurement of MSK pain in children and adolescents

From a measurement perspective, the need to consider assessment of pain in children in a different manner to adults is well-accepted^70^ ^,^ ^71^. However, there are few validated instruments to measure MSK conditions and consequences in children and adolescents. As is the case in adults, it is important to distinguish between trivial and consequential MSK pain and therefore simple prevalence estimates should be supplemented by information about frequency, intensity, disability and consequences^72^.

The largest population based study; The Health Behaviour in School-aged Children (HBSC) study from the World Health Organization^73^ uses the HBSC Symptoms Checklist. This checklist has questions about the frequency of ‘backache’ but no reference to pain intensity or other types of MSK problems. The same frequency categories have been used in other studies to report both spinal and extremity pain, either with the use of a mannequin^74^, or in combination with region-specific questions^22^ ^,^ ^75^. The Young Spine Questionnaire (YSQ) has been developed to include the three regions of the spine and includes questions on intensity and frequency. The YSQ has been validated in 12-14 year old Danish children^76^, but not yet in other samples and cultures.

Studies have also used a series of questions to identify the consequences of back pain^35^ ^,^ ^77^; these questions may be useful individually in description of specific impacts and also collectively as an indicator of severity. The questions relate to seeking of professional care, medication usage, absence from school or work, interference with normal activities, and interference with physical activities. A newly developed Teen Nordic Musculoskeletal Screening Questionnaire (TNMQ-S) also includes the impact on school and/or work attendance and on sporting and/or recreational activity participation as measures of severity. This questionnaire has shown good reliability and validity in a preliminary investigation of its measurement properties in Canadian teenagers^78^.

However, self-report questionnaires can only be used in literate children, so assessment of pain in younger children necessarily involves interaction with both children and their parents. This introduces complexity as it has been shown that the agreement between parent and child ratings is generally poor, but varies in relation to severity of the condition and the outcomes measured^79^ ^-^ ^81^. Therefore, besides reporting intensity, frequency and consequences of MSK pain, studies should describe the degree of parents’ involvement in data collection. See Box 6 for an overview of issues related to measurement.

**Box 6:** Measurement.

What we know • Pain and impact of pain can be measured using relatively simple questionnaires in literate children. • Parent’s and children’s pain reports cannot be used interchangeably. What we need • Measures that draw out the distinction between trivial and consequential MSK problems. • Further validation and cross-cultural adaptation of the YSQ and TNMQ-S. • Development of questionnaires to measure extremity complaints.

## Conclusion

Despite concerning data regarding the prevalence, impact and long-term consequences of musculoskeletal pain in children and adolescents, the field has not been subject to a concerted and systematic research effort. As a consequence there are major gaps in our understanding of these conditions, which leaves clinicians charged with treating young people with little empirical evidence to help guide their management decisions. There is particular need for research to better elucidate the clinical course of MSK pain conditions in children and adolescents, research to enable identification of those at risk of consequential pain and disability, studies to draw out the nature of the relationships between MSK pain and other adverse health risk factors and investigation into measurement issues. The body of research into MSK pain in adults is complicated by studies of poor methodological quality and inconsistent terminology and measurement, it is hoped that we can learn from this and generate a more robust, reliable and comparable body of evidence for children and adolescents.
